# Respiratory chain complex III deficiency due to mutated *BCS1L*: a novel phenotype with encephalomyopathy, partially phenocopied in a *Bcs1l* mutant mouse model

**DOI:** 10.1186/s13023-017-0624-2

**Published:** 2017-04-20

**Authors:** Saara Tegelberg, Nikica Tomašić, Jukka Kallijärvi, Janne Purhonen, Eskil Elmér, Eva Lindberg, David Gisselsson Nord, Maria Soller, Nicole Lesko, Anna Wedell, Helene Bruhn, Christoph Freyer, Henrik Stranneheim, Rolf Wibom, Inger Nennesmo, Anna Wredenberg, Erik A. Eklund, Vineta Fellman

**Affiliations:** 1 Division of Pediatrics, Department of Clinical Sciences, Lund University, 221 84 Lund, Sweden; 2 Folkhälsan Research Center, Helsinki, Finland; 3 Astrid Lindgren Children’s Hospital, Karolinska University Hospital, Stockholm, Sweden; 4 Faculty of Medicine, University of Helsinki, Helsinki, Finland; 5 Mitochondrial Medicine, Department of Clinical Sciences, Lund University, Lund, Sweden; 6 Department of Pathology, Regional Laboratories, Region Skåne, Lund, Sweden; 7 Division of Clinical Genetics, Department of Laboratory Medicine, Lund University, Lund, Sweden; 8 Centre for inherited Metabolic Diseases, Karolinska University Hospital, Stockholm, Sweden; 9 Department of Molecular Medicine and Surgery, Karolinska Institutet, Stockholm, Sweden; 10 Department of Medical Biochemistry and Biophysics, Karolinska Institutet, Stockholm, Sweden; 11 Max Planck Institute Biology of Ageing—Karolinska Institutet Laboratory, Division of Metabolic Diseases, Department of Laboratory Medicine, Karolinska Institutet, Stockholm, Sweden; 12 Department of Molecular Medicine and Surgery, Science for Life Laboratory, Center for Molecular Medicine, Karolinska Institutet, Stockholm, Sweden; 13 Department of Pathology, Karolinska University Hospital, Stockholm, Sweden

**Keywords:** Mitochondrial disorder, Respiratory chain, Respirometry, Assembly factors, Blue native gel electrophoresis, Encephalopathy, Hepatopathy, Microglia, Barrel cortex

## Abstract

**Background:**

Mitochondrial diseases due to defective respiratory chain complex III (CIII) are relatively uncommon. The assembly of the eleven-subunit CIII is completed by the insertion of the Rieske iron-sulfur protein, a process for which BCS1L protein is indispensable. Mutations in the *BCS1L* gene constitute the most common diagnosed cause of CIII deficiency, and the phenotypic spectrum arising from mutations in this gene is wide.

**Results:**

A case of CIII deficiency was investigated in depth to assess respiratory chain function and assembly, and brain, skeletal muscle and liver histology. Exome sequencing was performed to search for the causative mutation(s). The patient’s platelets and muscle mitochondria showed respiration defects and defective assembly of CIII was detected in fibroblast mitochondria. The patient was compound heterozygous for two novel mutations in *BCS1L*, c.306A > T and c.399delA. In the cerebral cortex a specific pattern of astrogliosis and widespread loss of microglia was observed. Further analysis showed loss of Kupffer cells in the liver. These changes were not found in infants suffering from GRACILE syndrome, the most severe *BCS1L*-related disorder causing early postnatal mortality, but were partially corroborated in a knock-in mouse model of BCS1L deficiency.

**Conclusions:**

We describe two novel compound heterozygous mutations in *BCS1L* causing CIII deficiency. The pathogenicity of one of the mutations was unexpected and points to the importance of combining next generation sequencing with a biochemical approach when investigating these patients. We further show novel manifestations in brain, skeletal muscle and liver, including abnormality in specialized resident macrophages (microglia and Kupffer cells). These novel phenotypes forward our understanding of CIII deficiencies caused by *BCS1L* mutations.

**Electronic supplementary material:**

The online version of this article (doi:10.1186/s13023-017-0624-2) contains supplementary material, which is available to authorized users.

## Background

Mitochondrial diseases are due to mutations in nuclear or mitochondrial genes encoding proteins directly or indirectly involved in oxidative phosphorylation (OXPHOS) or other important mitochondrial functions [1, 2]. An important subgroup amongst these disorders is the complex III (CIII) deficiencies (ubiquinol:ferricytochrome c oxidoreductase deficiency; cytochrome bc_1_ complex deficiency) [3]. CIII disorders have long been considered uncommon since traditional investigations for mitochondrial disease, including muscle biopsy (looking for ragged red fibers or cytochrome C oxidase (COX) negative fibers) and routine spectrophotometric methods for OXPHOS activity, do not necessarily reveal these deficiencies [4]. CIII catalyzes the transfer of electrons from reduced Coenzyme Q10 to cytochrome c, with the subsequent transfer of protons across the inner membrane of the mitochondria. It is a homodimer in which each monomer contains eleven subunits; two core proteins (encoded by *UQCRC1* and *UQCRC2*, respectively), three electron-transferring proteins with prosthetic groups (cytochrome b, cytochrome c_1_ and Rieske iron-sulfur protein (RISP) encoded by *MT-CYB*, *CYC1* and *UQCRFS1*, respectively) and six low molecular weight accessory proteins (encoded by *UQCRH*, *UQCRB*, *UQCRQ*, *UQCR10*, *UQCR11*, respectively, plus the N-terminal part of the RISP encoded by *UQCRFS1*) [5]. The assembly of this eleven-subunit complex requires the presence of chaperones/facilitating proteins not present in the functional mature protein, including the proteins encoded by *LYRM7* [6]*, TTC19* and *BCS1L* [5].

The BCS1L protein is required for the insertion of the RISP into the CIII pre-complex dimer (pre-CIII_2_). This step completes the structure of the mature, catalytically active complex. The corresponding protein in yeast, bcs1, is well characterized and has been shown to transport the RISP from the matrix of the mitochondria, where it has acquired its 2Fe-2S cluster, to the intermembrane space, where it assembles with the pre-CIII [7]. BCS1L is phylogenetically conserved and homologs are found in all eukaryotic genomes.

Diseases caused by *BCS1L* mutations range from the mild Björnstad syndrome, with brittle hair (*pili torti*) and sensorineural hearing loss [8] to the fatal GRACILE syndrome [9]. Several other phenotypes have been described that range in-between these conditions [10–13]. The GRACILE syndrome, an acronym for Growth Restriction, Aminaciduria, Cholestasis, Iron overload, Lactacidosis, and Early death, is due to a specific homozygous mutation so far only found in the Finnish population (c.232A > G; p.Ser78Gly) [14], with over 40 known cases. Since these patients die early in life, little is known about their psychomotor development. However, in GRACILE-like patients, and other patients with *BCS1L* mutations, encephalopathy, together with tubulopathy and liver disease are common features. In total, less than 100 patients have been described worldwide with conditions attributed to mutations in this gene. Knock-in mice, carrying the same missense mutation as the GRACILE syndrome patients, develop a phenotype that is similar to that seen in neonates and thorough analysis of the renal and hepatic pathologies have been published [15, 16].

We here describe two novel mutations in the *BCS1L* gene in a patient with a severe phenotype involving minimal psychomotor development, pronounced muscular hypotonia, aminoaciduria, growth restriction and premature death. The necropsy revealed specific changes in the brain (e.g. astrogliosis) that also were seen at P150 in an animal model of GRACILE syndrome (but not at P30). Similar changes were, however, not seen in the brains of GRACILE patients. The data suggests that there are temporally specific changes in the course of BCS1L deficiency. Furthermore, the patient exhibited hypomicrogliosis and had fewer Kupffer cells (KCs) suggesting a specific deficiency in yolk sac derived macrophages. Our findings further extend the phenotypic expression of this subtype of CIII deficiency.

## Methods

### Platelet respirometry

The patient blood samples were collected in K_2_EDTA tubes (Vacutainer, BD, Franklin Lakes, USA) via venous puncture. As control samples, blood from healthy children undergoing anesthesia for minor elective surgery was used (after written informed consent from their guardians was obtained). Platelets were isolated with consecutive centrifugation steps as previously described [17]. Respiration was measured in a high-resolution oxygraph in MiR05 buffer (Oxygraph-2 k Oroboros Instruments, Innsbruck, Austria) and data was recorded with DatLab software 4.3. (Oroboros Instruments). The substrate, uncoupler, inhibitor titration protocol has been published previously [17].

### Biochemical and morphological investigations in skeletal muscle

The patient was subjected to a percutaneous muscle biopsy taken from *m. tibialis anterior* under local anesthesia using a conchotome. Determination of mitochondrial adenosine triphosphate (ATP) production rate, respiratory chain enzyme activities, and citrate synthase activity was carried out as previously described [18].

For histologic examination of the skeletal muscle, standard techniques were used for light and electron microscopy [19]. Morphologic analyses of cryostat sections included staining with hematoxylin and eosin, modified Gomori trichrome, oil red O and periodic acid-Schiff reagent, and incubation for ATPase, NADH-tetrazolium reductase (NADH), succinate dehydrogenase (SDH), cytochrome C oxidase (COX), and combined COX/SDH.

### Cell culture

A skin biopsy was taken from the patient under local anesthesia; fibroblasts were set up and propagated in DMEM/F12 supplemented with 10% fetal calf serum, 1% glutamine and penicillin/streptomycin according to our local routine clinical protocol. The fibroblasts were stored in liquid nitrogen until usage. Fibroblasts from a patient without a mitochondrial disorder were similarly obtained and used as control cells along with fibroblasts obtained from the umbilical cords of two healthy term newborn infants.

### Protein analyses

For Blue Native PAGE (BN PAGE) analysis, mitochondria were prepared from fibroblasts and frozen for further analysis as previously described [20]. The protein concentration was estimated using NanoDrop (Thermo Scientific, NanoDrop Products, Wilmington, DE). Each sample (15 μg per well) was run on a NativePAGE Novex 4–16% Bis-Tris gel (Thermo Scientific) and blotted to PVDF membrane using Iblot equipment (Invitrogen, Carlsbad, CA). After blocking in 5% dry milk the blots were incubated with antibodies detecting BCS1L (Abnova, Taipei, Taiwan), two subunits of CIII (RISP, MS 305; CORE1, MS 303, Mitoscience, Eugene, OR, USA), complex IV (CIV) (subunit Va; MS 409, Mitoscience), complex II (CII) (30 kDa IP; MS 203, Mitoscience) and complex I (CI) subunit NDUFV1 (Sigma Aldrich, Stockholm, Sweden).

For Western Blot analysis snap-frozen liver autopsy samples or pelleted fibroblasts were homogenized in cold lysis buffer (50 mM Tris-HCL pH 7.4, 150 mM NaCl, 1% Triton X-100, 0.5% Na-deoxycholate, 0.1% SDS, 25 mM NaF, and 1 mM EGTA) containing protease inhibitor mix (Roche Complete Mini, Mannheim, Germany), and cleared by centrifugation (15 000 x *g* at 4 °C). Equal amounts (10–20 μg) of reduced and denatured protein were run on Tris-glycine 4–20% gels (Bio-Rad Laboratories Inc. Hercules, CA, USA). The resolved proteins were then transferred onto PVDF membrane using the Trans-Blot Turbo semi-dry system (Bio-Rad). Amount of protein transferred onto membranes was visualized with Ponceau S staining and inspected for equal loading and protein pattern. The membranes were probed with antibodies raised against the following: BCS1L (HPA037701, Atlas Antibodies Ltd.), RISP (see above), CORE1 (see above), NDUFA9 (MS111, Mitosciences), SDHB (ab14714, Abcam, Cambridge, Great Britain), COXI (MS404, Mitosciences), VDAC1/porin (ab154856, Abcam). Horseradish peroxidase-conjugated secondary antibodies (Cell Signaling Technology, Danvers, MA) and enhanced chemiluminescence (ECL plus, Thermo Scientific, Waltham, MA) or ECL Femto (Thermo Scientific) (BCS1L detection) were used for detection. The luminescence was recorded with ChemidocMP CCD imager (Bio-Rad). Sample preparation and western blot analyses were repeated at least twice with identical results.

### DNA and RNA isolation

Genomic DNA from the patient and her parents was isolated from EDTA-blood using the QIAamp DNA Midi Kit (Qiagen, Sollentuna, Sweden). For RNA analysis, blood was collected in PAXgene Blood RNA Tubes (Qiagen) and total RNA was isolated using the PAXgene Blood RNA Kit (Qiagen). Total RNA was isolated from patient and control fibroblasts using NucleoSpin RNA kit (Macherey-Nagel) with an on-column DNAse digestion.

### Whole exome sequencing and bioinformatics

Whole exome sequencing on genomic DNA samples from the patient and her parents was performed as described previously [21], followed by in-house computational analysis, using the mutation identification pipeline [21]. Only variants in genes known to cause a metabolic disorder were analyzed. The list of genes (dbCMMS) is published on the following site: http://karolinska.se/globalassets/global/kul/cmms/dbcmms.v1.1.pdf. The splice prediction tools SPIDEX [22] and NetGene2 were used for analyzing the synonymous mutation in *BCS1L*.

### Molecular analysis of BCS1L

Sanger sequencing of the two mutations in *BCS1L* was carried out following PCR amplification of genomic DNA using the following M13-tagged primers: BCS1L_F:AGACTTCGTACCTTCAGCAT and BCS1L_R:GCTGTGCCAAACA GCTTCCT. RT-PCR was performed on isolated RNA using the IScript cDNA Synthesis Kit (Bio-Rad) and the following M13-tagged primers: BCS1LcDNA_F:CCTTTCAAGATGCCACTTTC and BCS1LcDNA_R:ACTGCTCT TTCCGCAACCAG. Subsequent sequencing of the PCR products was carried out with M13 primers using the BigDye version 3.1 sequencing kit (Applied Biosystems) on a 3500xl Genetic Analyzer (Applied Biosystems) with alignment to the reference sequence NM_004328. A quantitative PCR (qPCR) assay using 7 gene-specific amplicons encompassing the coding exons 3–9 of the *BCS1L* gene was performed by Centogene, Rostock, Germany. For additional verification of the mutation analysis, cDNA was prepared from DNAse-treated total RNA, isolated from patient and control fibroblasts, using RevertAid reverse transcriptase and random hexamers (Thermo Scientific). Reactions without reverse transcriptase were included as controls. Full *BCS1L* coding region or shorter fragments spanning exons 3 and 4 were amplified using Phusion Hot-Start polymerase (Thermo Scientific) and sequenced. For the c.306A > T aberrantly spliced transcript-specific RT-PCR, Phusion polymerase buffer GC was used, the reactions were amplified for 36 cycles, and reaction products run on 2% agarose-TBE gels with Midori Green (Nippon Genetics Europe) for detection.

### Autopsy tissue specimen

A routine autopsy was performed on the deceased and tissue samples were fixed in paraformaldehyde for histology per clinical routine. Specimens from liver, heart muscle and brain were directly snap frozen at -80 °C for future genetic and biochemical analysis. The brain was formalin fixed en bloc. Previously obtained and prepared brain tissue samples from infants who died from GRACILE syndrome due to the homozygous c.232A > G mutation in *BCS1L* (*n* = 5 [20]) and of four infants (aged 8–17 months) who died of other causes than mitochondrial disease (pulmonary stenosis, cerebellar vascular anomaly, congenital heart defect or SIDS) were used for comparison. Snap frozen liver specimens from two diseased infants were obtained via the Department of Pathology, Helsinki University Central Hospital, Helsinki, Finland.

### Animal maintenance

Mice harboring the *Bcs1l* ^c.232A>G^ mutation [15] were in the C57BL/6JCrl genetic background. In this strain the homozygous mice survive up to approximately 6 months. They were maintained at the animal facilities of University of Helsinki, Finland, in individually ventilated cages with 12 h light/dark cycle at 22 °C. Chow (Harlan Teklad 2018) and water was available *ad libitum*.

### Histological processing

*Bcs1l* ^c.232A>G^ and control mice were perfused with 4% paraformaldehyde at postnatal day 150 (P150; *n* = 6 per genotype). Brains were immersion fixed in 4% paraformaldehyde in 0.1 M sodium phosphate buffer pH 7.4 for 48 h. Half the brain was cryoprotected in 30% sucrose/0.05% sodium azide in 50 mM Tris buffered saline (TBS) and 40 μm frozen coronal sections were cut through cerebrum, while cerebella were cut sagittally, and stored in cryoprotectant solution (30% ethylene glycol/15% sucrose/0.05% sodium azide in TBS). The other half of the brain was cast in paraffin. Paraffinated samples (patient and mouse brain and liver) were cut into 5 μm sections.

### Immunohistochemistry

Free-floating cryosections were stained as previously described [23]. Briefly, sections were incubated for 15 min in 1% hydrogen peroxide in TBS and blocked for 2 h with 15% normal serum/0.3% Triton X-100 in TBS (TBS-T). Primary antibody diluted in 10% normal serum in TBS-T was incubated overnight at 4 °C and biotinylated secondary antibody (Vector Laboratories, Burlingame, CA, USA) for 2 h. Sections were incubated for 2 h in Vectastain avidin-biotin-peroxidase complex (Vectastain Elite APC kit, Vector Laboratories) and immunoreactivity visualized by a standard diaminobenzidine-hydrogen peroxide reaction (Sigma). Sections were mounted onto gelatine-chrome alum-coated microscope slides (Southern Biotechnology Associates, Inc., Birmingham, AL, USA), air-dried overnight and passed through a graded series of alcohols before clearing in xylene and coverslipping with DPX mounting media (Sigma).

Paraffin sections were dewaxed with xylene and descending series of alcohol and incubated for 5 min in 5% hydrogen peroxide in PBS. Antigen retrieval was performed by lightly boiling sections in 10 mM sodium citrate, pH 6.0 for 20 min, followed by cooling at RT for 1 h. Sections were blocked with 5% normal serum PBS and primary antibody diluted in 1% normal serum in PBS was incubated overnight. Secondary antibodies were diluted to 1% FCS in PBS and incubated for 30 min. Nuclei were counter stained with 1 ng/ml Hoechst 33258 (Thermo Fischer Scientific, Waltham, MA, USA). Primary antibodies were raised against the following: GFAP (Z0334, DAKO, Agilent Technologies, Inc., Santa Clara, CA, USA), RISP (HPA041863, Sigma), IBA1, (019-19741, Wako Chemicals GmbH, Neuss, Germany), CD11b (ab133357, Abcam).

### Image analysis

All microscopic images were taken with AxioCam HRc (Carl Zeiss AG, Oberkochen, Germany). Cortical images were taken as several individual overlapping images and merged together using Photomerge in *Adobe Photoshop CS4* software (Adobe Systems Inc., San Jose, USA).

## Results

### Patient description

The girl was the first child to unrelated, healthy parents. The mother had no history of missed abortions/miscarriages. The girl was born after an uneventful pregnancy after labor induction in the 42nd gestation week. Due to a pathological cardiotocogram (CTG), vacuum extraction was used to assist delivery (birth weight 3500 g, birth length 55 cm, head circumference 36.8 cm; Apgar 7-8-8). She was pale and hypotonic and respiratory assistance (continuous positive pressure ventilation) was needed for 20 minutes. At 2 h lactic acidemia (pH 7.0, lactate 8.2 mmol/L, base excess -10 mmol/L) was noted, which persisted over the following days. At day 4, ultrasound and MRI of the head revealed a left sided grade III intraventricular hemorrhage (IVH) without ischemic changes. The parallel MR spectroscopy was deemed normal. No cause of the hemorrhage could be established. An increasing head circumference and signs of hydrocephalus complicated the clinical course, but she never needed surgical intervention.

The muscular hypotonia persisted and there was feeding difficulty requiring a feeding tube over the first month. The psychomotor development was severely affected and at a neurological examination at 4.5 months the development corresponded to 6 weeks. There was a general hypotonia, the movements of the legs and arms was largely reduced and stereotypical in quality. Eye contact could not be established and she had almost no sound production. She however reacted to sound and light/dark changes. She made no intention to turn over from back to belly and reverse. Spasticity was noted in the legs. A neurometabolic screen was initiated (see below) since the symptoms were considerably more severe than expected. A repeat MRI showed a progressive loss of the white matter and a secondary enlargement of the ventricles. An adequate spectroscopy could not be performed due to the leucodystrophic changes. Over the following months development was largely absent. She was able to swallow formula, but not in sufficient amounts and developed severe growth failure. After a discussion in the Ethical Committee of the hospital, the parents’ request not to put nasogastric feeding tube or gastrostomy was granted (due to the dismal prognosis). The girl passed away at 13 months of age. The autopsy revealed severe wasting of the organs with a body weight of 4850 g. The final cause of death was probably myocardial infarction.

### Biochemical work-up

Due to the inexplicable deterioration of the patient’s development, not fully explained by the intraventricular hemorrhage, an extensive biochemical work-up was initiated. No abnormalities were noted in the free carnitine level, acylcarnitines, organic acids, glycosaminoglycans and other complex oligosaccharides and peroxisomal screening tests (phytanic acid, very long chain fatty acids and plasmalogens). The plasma aminogram was normal, however, an unspecific increase in the level of urine amino acids was noted, indicating a potential mitochondrial defect (data not shown). Lactate in cerebrospinal fluid was increased (3.4 mmol/L). Analysis of cerebrospinal fluid further revealed a massive increase in the marker of brain damage neurofilament light protein (NFL; 32600 ng/L, reference value <380) indicating a progressive neurodegenerative disease.

### Mitochondrial work-up

**Fig. 1:**
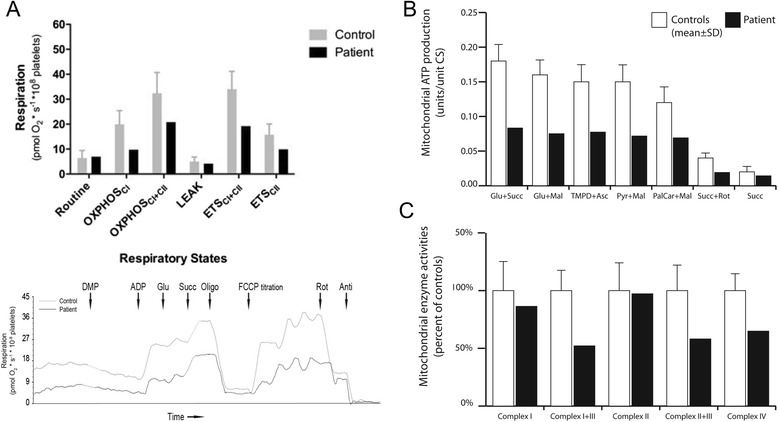
Respirometry indicates mitochondrial disease. **a** *Upper panel*. Platelet mitochondrial respiration in patient and controls (*n* = 13; 1 month - 3 years; mean values ± SD). Respiration is expressed as pmol O_2_/s/10^8^ platelets. Induced respiratory states and activated respiratory complexes are defined on x-axis. Routine, endogenous basal respiration of intact platelets; following cell membrane permeabilization: OXPHOS_CI_, phosphorylating respiration (OXPHOS) in presence of ADP and CI substrates (pyruvate, malate and glutamate); OXPHOS_CI+CII_, respiration in presence of ADP, CI and CII (succinate) substrates; LEAK, oligomycin-inhibited non-phosphorylating basal respiration (in presence of CI and CII substrates); ETS_CI+CII_, uncoupler (FCCP)-induced non-phosphorylating maximal capacity of the electron transport system (ETS); ETS_CII_, maximal non-phosphorylating CII-related respiration. *Lower panel*: representative traces of the substrate, uncoupler, inhibitor titration protocol of the patient platelets and one of the control samples. Consecutive additions of digitonin (for permeabilization) plus malate and pyruvate (DMP), ADP, glutamate (Glu), succinate (Succ), oligomycin (Oligo), uncoupler titration using FCCP, rotenone (Rot) and finally antimycin (Anti). **b** Muscle mitochondrial function in patient and controls (*n* = 11; 0–5 years; mean values ± SEM). Mitochondrial ATP production with the substrate combinations glutamate + succinate, glutamate + malate, TMPD + ascorbate, pyruvate + malate, palmitoyl-L-carnitine + malate, succinate + rotenone and succinate only. **c** Activities for the respiratory chain enzymes (NADH-coenzyme Q reductase (complex I), NADH-cytochrome c reductase (complex I + III), succinate dehydrogenase (complex II), succinate-cytochrome c reductase (complex I + III) and cytochrome c oxidase (complex IV). All activities are expressed relative to the controls. Mitochondrial ATP production and the respiratory chain enzyme activities were determined as units/unit citrate synthase activity in isolated mitochondria

**Fig. 2:**
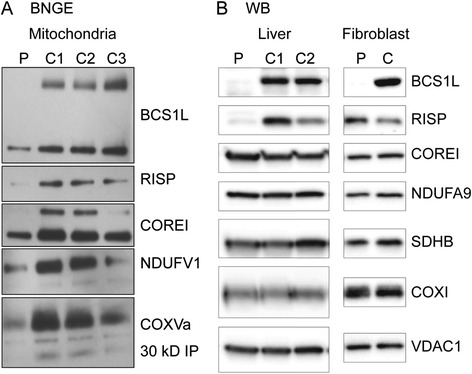
Blue Native PAGE and Western blot analysis of patient fibroblasts and liver. **a** The presence of respiratory chain complexes I-IV (CI-CIV), CIII assembly and BCS1L protein from the patient (P) and controls (C1-C3) were analyzed in fibroblast mitochondria using BN PAGE technique. C1 and C2 are fibroblasts from umbilical cords from healthy pregnancies, C3 are fibroblasts from a child with no symptoms of mitochondrial disease. Monomers (lower band) and oligomers (upper band) of BCS1L were detected using antibodies raised against this protein. CIII was investigated using antibodies directed against the two CIII subunits RISP (mature CIII) and CORE1 (lower band pre-CIII, upper band mature CIII). CI was assessed using an antibody against NDUFV1. Antibodies against 30 kDa IP and cytochrome c oxidase subunit Va (COXVa) were used to detect CII and CIV, respectively. The data shows a clear reduction of mature CIII complexes (with incorporated RISP) in the patient cells and loss of BCS1L protein. The amount of the other complexes (CI, CII and CIV) in patient cells and C3 is less than in C1 and C2, but the ratios of the individual complexes are similar in-between the samples. **b** Western blot analysis of homogenates from liver and fibroblasts from the patient (P) and two controls (C1 and C2). A loss of BCS1L protein and clear reduction in liver RISP is seen in accordance with BCS1L deficiency

In intact platelets, routine respiration (platelets using endogenous substrates only) was similar between the controls and the patient. After plasma membrane permeabilization and saturation with CI linked substrates (malate, pyruvate and glutamate), and subsequently the CII substrate succinate, oxidative phosphorylation (OXPHOS) displayed reduced capacity compared to controls indicating a respiratory dysfunction of CI or downstream thereof. Furthermore, non-phosphorylating CII-linked respiration, revealed by the addition of the CI inhibitor rotenone, was lower as compared to controls. Taken together, the results indicate a limitation in electron transport downstream of CII (Fig. 1a). Analysis of mitochondria isolated from muscle showed decreased activities in several complexes (CI + CIII, CII + CIII, CIV) and in overall ATP production (Fig. 1b and c). Standard BN PAGE techniques were used to assess respiratory chain organization (Fig. 2a, Additional file 4: Figure S3). The quantity of fully assembled CIII was investigated using antibodies directed against two CIII subunits (RISP and CORE1). There was an almost complete lack of fully assembled CIII and BCS1L (both oligomer and monomer) in patient cells. The quantities of the other complexes (CI, CII and CIV) were lower in patient cells and in cells of control number 3 (C3), compared to the other controls (C1 and C2), but the ratios between the complexes were within normal variation.

In Western blot analyses of cell lysates from liver and fibroblasts, the BCS1L protein was also completely missing in both tissues, whereas RISP was reduced in liver extracts, but present in fibroblasts (Fig. 2b).

Overall the data suggests that the BCS1L protein is largely absent causing a deficient incorporation of RISP into the pre-CIII and hence loss of functional CIII complexes.

### Genetic analysis

**Fig. 3:**
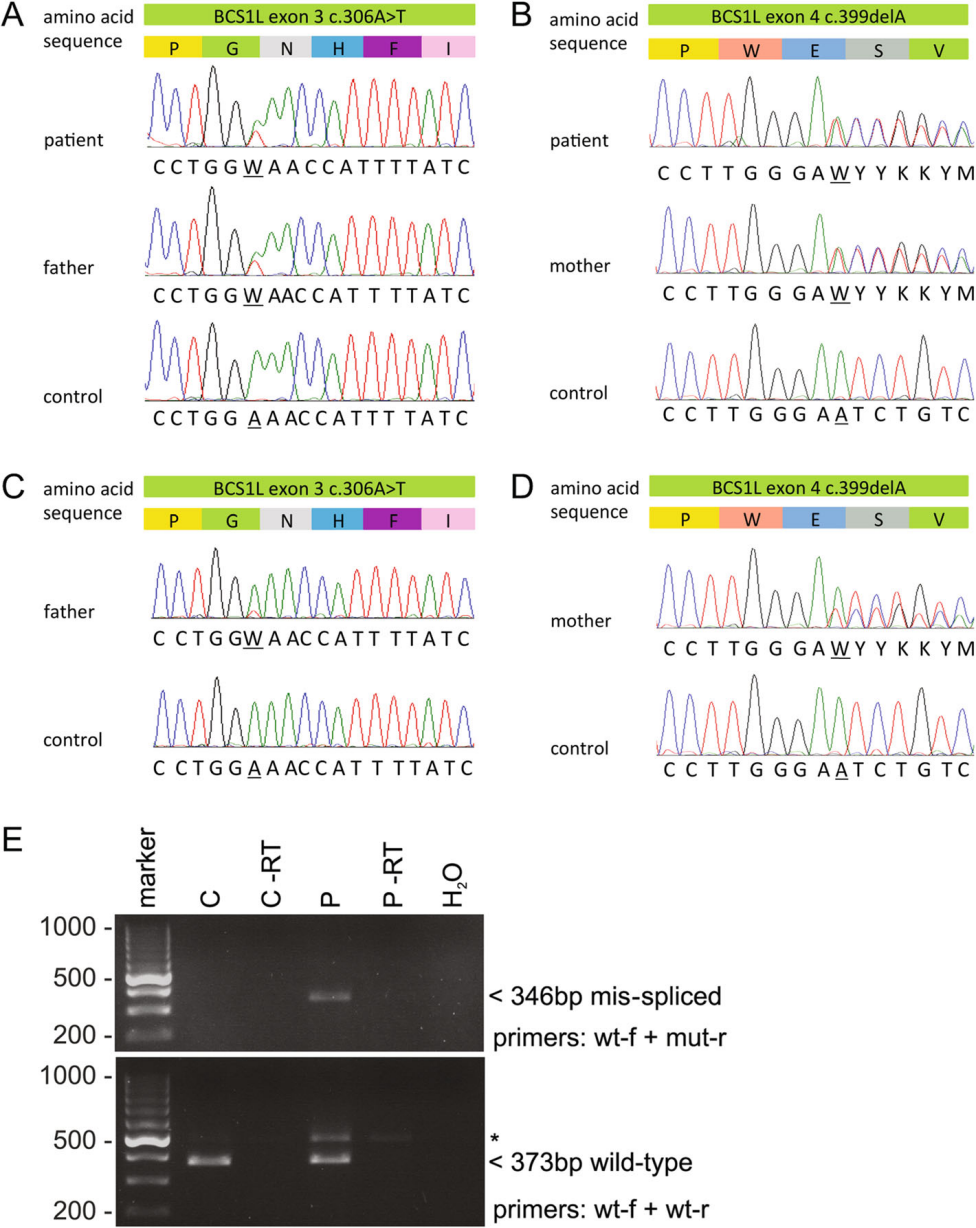
*BCS1L* mutations in patient and parents. Sanger sequencing of the *BCS1L* gene in the patient, parents and control genomic DNA. **a** c.306A > T inherited from the father and (**b**) c.399delA inherited from the mother. **c** Sequencing of cDNA from the father showed the wild-type transcript and a small amount of the correctly spliced transcript carrying the c.306A > T mutation, whereas (**d**) sequencing of the mother’s cDNA shows expression of the transcript carrying the c.399delA mutation. **e** Transcript-specific RT-PCR analysis of the c.306A > T splice site mutation in patient and control fibroblasts. The upper gel shows a 346-bp fragment amplified from the patient (P) but not from the control (C) fibroblast cDNA, confirming the presence of incorrectly spliced transcript in the patient. The lower gel shows a 373-bp wild-type fragment amplified from both control and patients cDNA. Asterisk denotes a larger fragment likely from a partially spliced transcript retaining the 98-bp intron between exons 3 and 4. A fragment of similar size is also faintly detectable in the –RT (minus reverse transcriptase) control for the patient sample. H_2_O denotes a control PCR reaction without template

A genomic array analysis did not show any copy number variations (CNVs) of significance (data not shown). Whole exome sequencing was performed and the data was filtered using the CMMS panel (dbCMMSv1). Two single nucleotide variations (SNVs) were detected in the *BCS1L* gene, c.306A > T and c.399delA. The mutation c.399delA is not previously described, but analysis using several software programs (including SIFT, PolyPhen2) indicated that it would severely affect protein function. It causes a frameshift and introduces a premature stop codon after 25 amino acids (p.Glu133AspfsTer25). The c.306A > T is a synonymous mutation (p.Gly102=) and was therefore initially deemed non-pathogenic. Therefore, all coding exons of the *BCS1L* gene were analyzed using a quantitative PCR assay (qPCR) to exclude copy number variations (CNVs) not detected by the genomic array analysis. No CNVs were found. The c.306A > T mutation was therefore analyzed using the splice prediction tools SPIDEX and NetGene2, which suggested that it creates a cryptic splice site in exon 3. Use of this aberrant splice site predicts a frameshift (p.Asn103IlefsTer8) in exon 4 and hence a truncated protein. To investigate the effect of the c.306A > T mutation on splicing, and hence its potential pathogenicity, we analyzed total RNA extracted from the parents’ blood (as the patient was deceased and hence no more blood could be apprehended). The patient’s mother was a heterozygous carrier of c.399delA and the father was heterozygous for the c.306A > T mutation (Fig. 3a and b). Sequencing of the mother’s cDNA clearly showed equal quantities of the wild-type allele and the allele carrying c.399delA (Fig. 3d). However, sequencing of the father’s cDNA showed a predominance of the wild-type allele and low level of the correctly spliced transcript carrying the c.306A > T mutation (Fig. 3c). The incorrectly spliced, frameshifted transcript was not detected on chromatograms, suggesting that it is unstable in this cell type. To further assess the pathogenicity of the silent c.306A > T change RT-PCR analysis from patient and control fibroblasts was performed. Amplification and sequencing of the whole *BCS1L* coding region verified the presence of the frameshifted transcript from the allele carrying the c.399delA mutation, but the allele carrying the c.306A > T variant was correctly spliced, suggesting low level of the putative aberrantly spliced transcript. For a more sensitive detection, transcript-specific RT-PCR was performed using a reverse primer spanning the 16-bp deletion predicted by mis-splicing. RT-PCR amplified the predicted mutant fragment from patient RNA but not from control RNA, whereas a similar wild-type fragment was amplified from both (Fig. 3e). The fragment amplified from patient RNA was extracted from the gel and sequenced, which confirmed that it corresponded to the predicted mis-spliced transcript (Additional file 1: Figure S4). Thus, also the cryptic splice site created by the c.306A > T change and generating a frameshifted transcript was used in patient fibroblasts.

### Histology and macroscopical autopsy findings

#### Muscle

Microscopic analysis of the skeletal muscle showed many fibers with an enhanced staining for NADH, SDH and COX (Additional file 2: Figure S1A). In ATPase staining they seemed to be of type 1. In Gomori trichrome staining these fibers had an increased red staining but no classical ragged red fibers were present. There was also an increased lipid accumulation in the fibers seen in oil red O staining.

Electron microscopy showed scattered fibers, which contained numerous mitochondria and also increased amount of lipid droplets (Additional file 2: Figure S1B). No paracrystalline inclusions were found but some mitochondria had structural abnormality of cristae, such as circular cristae. The combined analyses were clearly indicative of mitochondrial disease, however, the pattern was not specific for a defined type.

#### Autopsy

The main macroscopic finding at the autopsy was a general wasting of the internal organs and paleness indicative of anemia. On the macroscopic level, the brain exhibited linear focal cortical damage (Additional file 3: Figure S2).

#### Brain

**Fig. 4:**
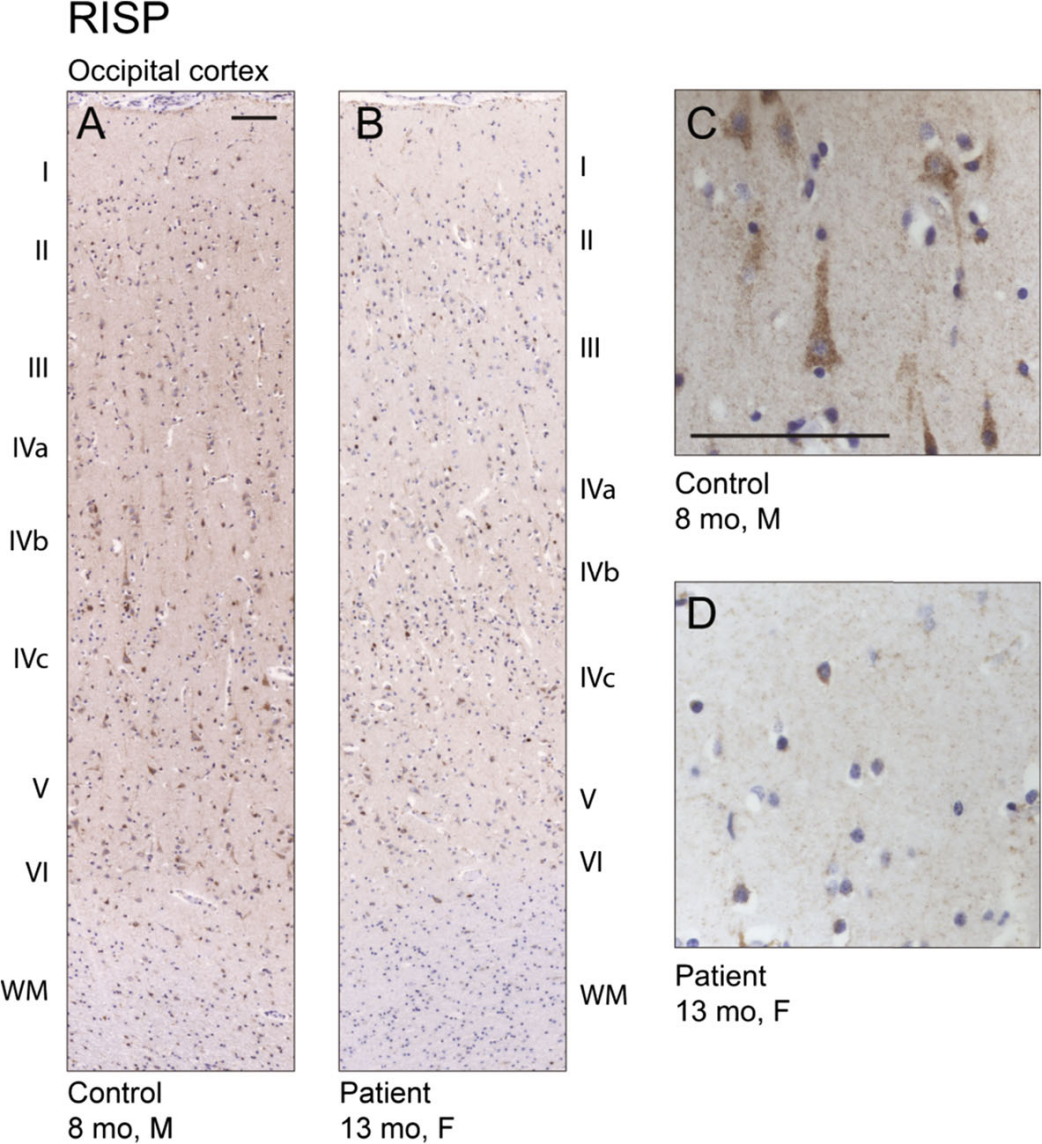
Reduced Rieske iron-sulfur protein (RISP) immunoreactivity in patient brain. **a** Immunostaining for RISP in the occipital cortex of control and (**b**) patient brain. Cytoplasmic localization of RISP in cortical neuronal cells in control (**c**) and reduced amount of RISP immunoreactivity in the patient brain (**d**). Scale bars 100 μm

**Fig. 5:**
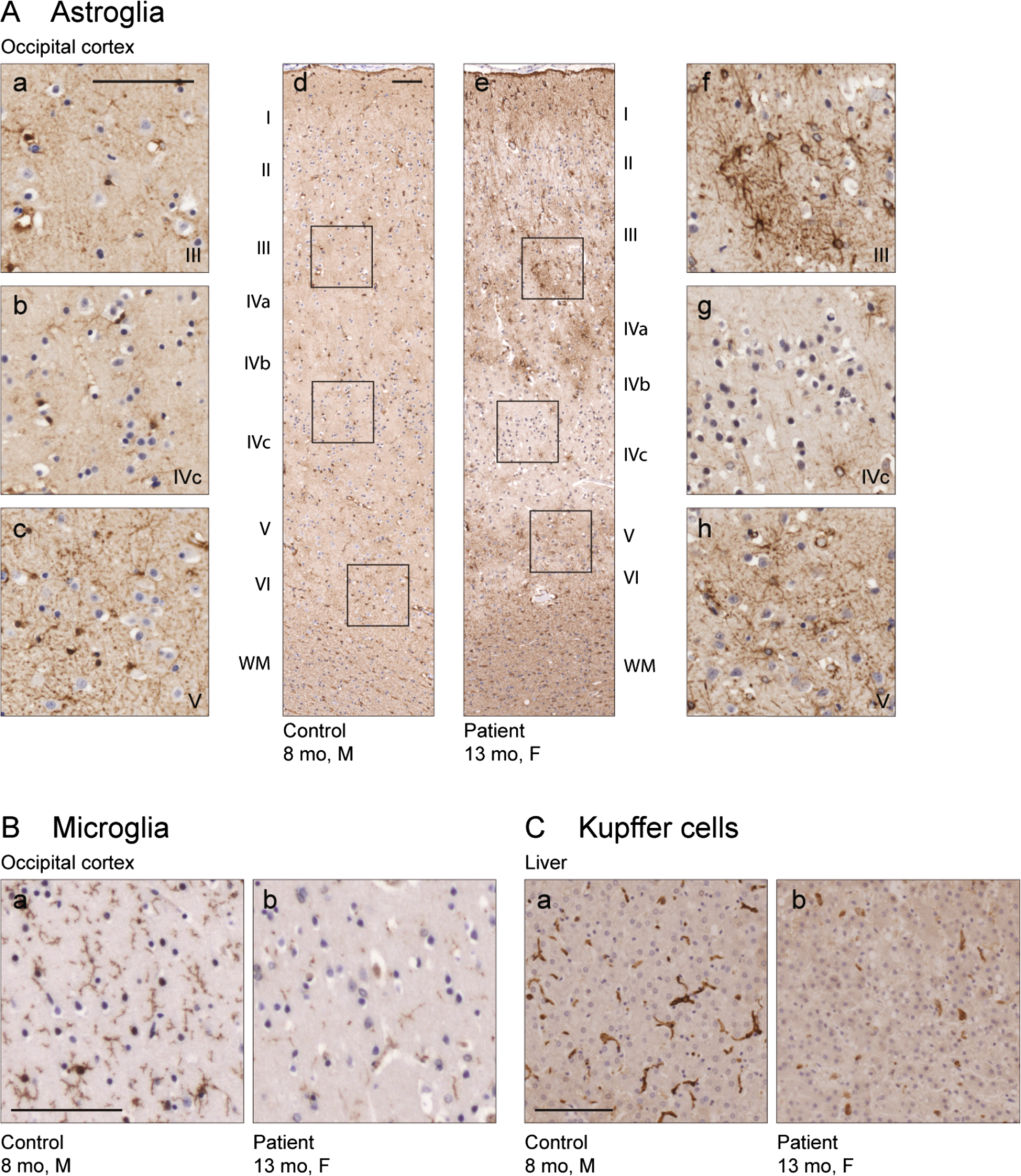
Patient brain and liver immunohistochemistry. (**a**) Increased immunoreactivity for the astroglial marker glial fibrillary acidic protein (GFAP) and change in the morphology of astroglial cells, two classical signs for astroglial activation, can be seen in the occipital cortex of the patient (*e*-*h*), but not in control brain (*a*-*d*). Activation is less pronounced in the lower part of the layer IV (*g*, *b* for ctrl), compared to the stronger activation in the upper part of the layer IV (*f*, *a* for ctrl) and layers V-VI (*h*, *c* for ctrl). The areas in the insets a-c and f-h are shown in figures d and e, respectively. (**b**) Immunostaining for the microglial marker IBA1 reveals loss of microglial cells and their processes in the cortex of the Lund patient (*a*) compared to the control (*b*). (**c**) Similar loss of Kupffer cells can be seen in the patient liver (*a*) and control (*b*). Scale bars 100 μm

There was a paucity of white substance in the patient brain in general, however the myelinization was deemed adequate. Immunohistochemical staining showed reduced RISP reactivity in the cerebral cortex compared to the children of similar age (Fig. 4). Astroglial activation was seen in several areas in the patient brain, being most distinctive in cerebral cortex and hippocampus. In all cortical areas studied, astrogliosis formed a striped pattern, in which layers III, upper part of IV, V and VI were clearly affected, while less reactive astrocytes were seen in layer II and lower part of the layer IV (Fig. 5a). Further, a clear reduction of microglial cells and their processes was noted in most of the brain regions, especially in cerebral cortex and cerebellum (Fig. 5b). The remaining microglial cells do not present with particularly activated phenotype. These findings were confirmed by using two different markers for microglia, IBA1 (Fig. 5b) and CD11b (data not shown).

Brains of five GRACILE patients were studied to see if they presented with similar findings as our patient. We could not observe any significant signs of astrogliosis, nor was the amount or phenotype of the microglial cells changed in these brains (data not shown).

#### Liver

Electromicroscopic analysis of liver mitochondria showed lack of identifiable cristae and osmiophilic depositions (data not shown), well in accordance with a mitochondrial disease, however the finding being an artifact could not be ruled out. Immunohistochemical staining with IBA1 and CD11b showed a marked reduction of KCs in the liver parenchyma of the patient, compared to the control individuals of similar age (Fig. 5c).

### Animal model histology

**Fig. 6:**
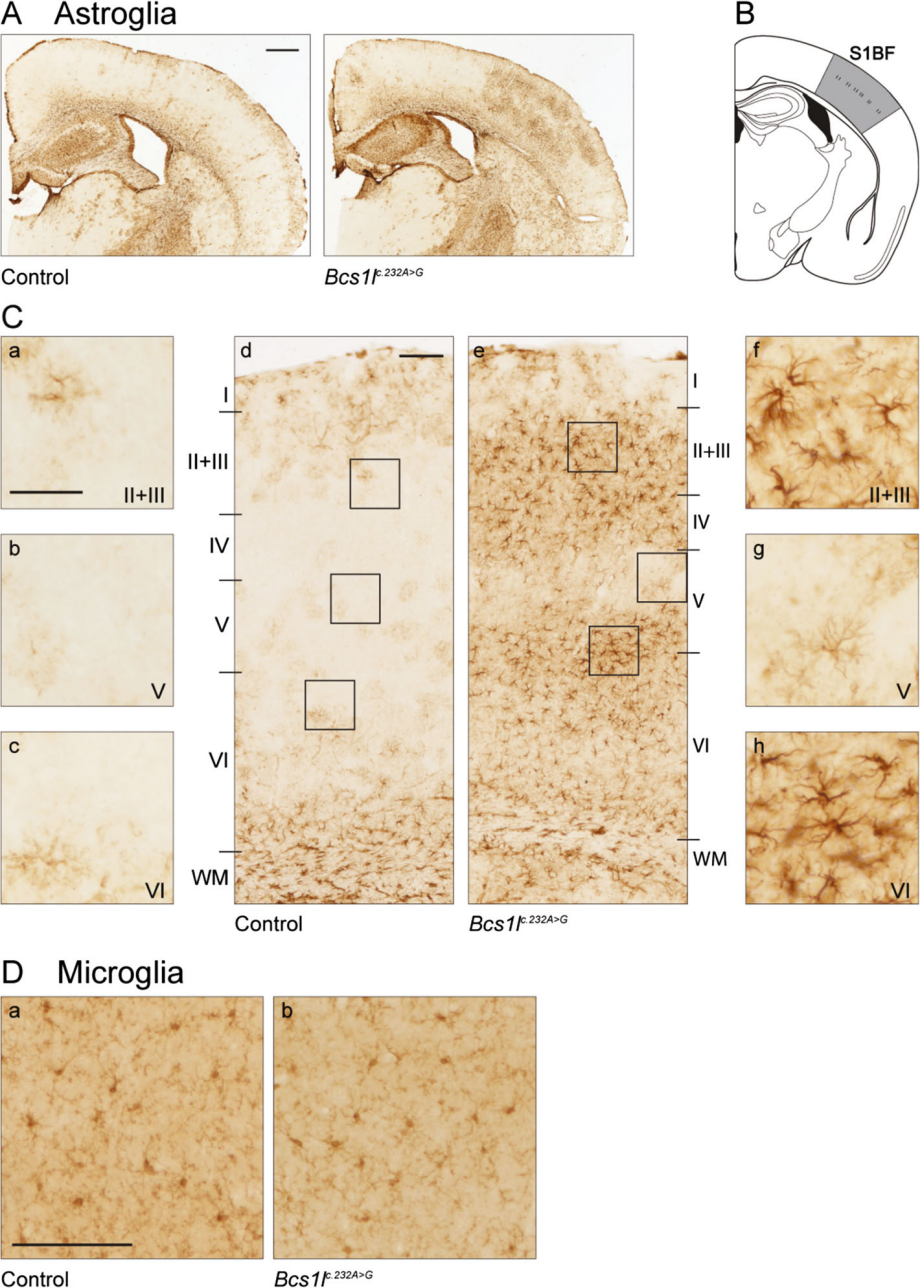
Local astroglial activation in the barrel field of primary somatosensory cortex of the homozygous *Bcs1l* ^c.232A>G^ mouse. (**a**) Immunostaining for the astroglial marker glial fibrillary acidic protein (GFAP) reveals localized astroglial activation in the barrel field of the primary somatosensory cortex (S1BF) of *Bcs1l* ^c.232A>G^ mouse, but not in control animals. (**b**) Schematic representation of the S1BF according to Paxinos and Franklin (2001) [36]. (**c**) Astroglial activation shows a pattern in which the upper part of cortical layer V is less affected (*g*, *b* for ctrl) compared to the more strongly affected layers II-IV (*f*, *a* for ctrl) and lower part of layer V and upper part of layer VI (*h*, *c* for ctrl). The areas in the insets a-c and f-h are shown in figures d and e, respectively. (**d**) No differences in the amount or the phenotype of the microglial cells were seen in the *Bcs1l* ^c.232A>G^ mouse compared to the control. Scale bars (**a**) 500 μm, (**b**) *d* and *e* 100 μm and *a*-*c*, *f*-*h* 50 μm, (**c**) 100 μm

The pathological changes in brain were also compared to the model for BCS1L deficiency, the homozygous *Bcs1l* ^c.232A>G^ mouse. Immunohistochemical analysis showed general mild astrogliosis throughout the brain. In contrast to that, substantial astroglial activation was found highly localized to the Barrel field of the primary somatosensory cortex (S1BF). Here the activation showed a strikingly similar pattern to the patient brain, with strong gliosis in layers II, III, IV and VI but preserved layer V (Fig. 6a-c). No signs of neurodegeneration were detected in the *Bcs1l* ^c.232A>G^ mouse brain. The volume of cerebral cortex and cerebellum was unchanged (data not shown), as was the thickness and the amount of neurons in the individual layers of S1BF (data not shown). No changes in the phenotype or amount of the microglial cells were observed (Fig. 6d).

## Discussion

Since mitochondrial genetics is complex, involving genes in both the nuclear and mitochondrial genomes, and the functions of many mitochondrial proteins are unknown or only partially characterized, investigation into the genetic cause of the mitochondrial disease in a given patient is often extensive and difficult. In modern clinical practice it usually involves next-generation sequencing [2] and a thorough biochemical work-up; the genetic data also often needs to be confirmed by analyzing gene products and metabolites. In our case, exome sequencing, where the raw data was filtered for genes previously described in mitochondrial disease, revealed one suspected pathogenic mutation in a mitochondrial gene; a deletion of one nucleotide (c.399delA) causing a frameshift (p.Glu133AspfsTer25) in *BCS1L*. Since this mutation is predicted to lead to severe protein truncation it was deemed very probably damaging. As the respiratory chain investigations in the patient’s muscle had revealed a complex III defect and due to the severe phenotype, the finding of a pathogenic mutation in *BCS1L* made it a strong candidate. We therefore analyzed the whole gene using qPCR of all coding exons, but no CNVs were detected. Hereafter, SNVs that were previously deemed non-pathogenic were analyzed *in silico* and the splice prediction tools SPIDEX and NetGene2 suggested the synonymous nucleotide exchange c.306A > T (p.Gly102=) could introduce an intra-exonic splice site whose use would produce a frameshifted transcript and thus be potentially pathogenic. According to *in silico* prediction the probability of use of the correct and aberrant cryptic splice sites was essentially the same (0.69 and 0.67, respectively), predicting about 50% of normally spliced transcript and full-length protein from this allele. This is in line with previous reports that have described truncating (loss-of-function) mutations only in combination with missense mutations (likely partial loss-of-function) in compound heterozygous patients [4]. In blood from the father (heterozygous carrier), the transcript from this allele was barely detectable suggesting decay of this message. Further, RT-PCR and sequencing analyses of patient fibroblasts confirmed that both correctly and incorrectly spliced, frameshifted transcripts are produced from this allele. These data did not allow quantitation of wild-type versus mutant transcript levels, but did show that mis-splicing takes place and, subsequently, total wild-type mRNA is inevitably reduced to below 50% leading to reduced BCS1L protein (as shown by BNGE and Western blotting) and disease manifestation. Our findings emphasize the importance of thorough analysis of SNVs, when the first filtered analysis of exome data does not reveal the cause of the disease.

Parallel to the genetic investigations we analyzed isolated mitochondria from patient fibroblasts, using BN PAGE analysis. This analysis was in accordance with BCS1L deficiency with a clear decrease in the formation of mature CIII from the existing pre-CIII, while the other complexes formed normally. It is known, however, that fibroblasts from GRACILE patients can show normal composition of complexes [20], why a normal BN PAGE analysis does not exclude BCS1L pathology. In the present patient, Western blot analysis of homogenates from liver and fibroblasts showed a clear decrease (almost absence) of BCS1L protein and, in liver, also a clear decrease in RISP. Taken together, these results prove the pathogenicity of the mutations identified in our patient [20].

In a recent review the phenotypes of the more than 20 different BCS1L mutations were categorized in three groups; purely visceral, pure encephalopathy and milder phenotypes [4]. In its most severe form, BCS1L deficiency causes GRACILE syndrome [9]. The phenotype of the current patient includes some similarities to this syndrome; *i.e.* a marked postnatal metabolic lactic acidosis, aminoaciduria indicating proximal tubulopathy, liver manifestation, and postnatal failure to grow. However, there are major differences: fetal growth was normal, the metabolic acidosis was reversible maybe due to the possibility to recruit energy fuel from glycogen and deposits in adipose tissue not present in the severely growth restricted GRACILE syndrome newborns. Further, the liver manifestation was very minor, no iron accumulation was found, the muscle and cerebral manifestations were the major findings present already in the neonatal period, and the survival was considerable longer. The oldest patient with GRACILE syndrome survived to 4 months and thorough neurohistological analysis of brains from these patients did not reveal any abnormalities [24, 25] as also verified in this study. In many mitochondrial disorders, however, neuropathological changes are evident. Some features of neuropathology seem to be shared across the spectrum of mitochondrial disorders, such as gliosis, spongiform degeneration, and neuronal loss [26] whereas others seem more specific, e.g. in Leigh syndrome where focal bilateral symmetrical lesions in the brainstem and basal ganglia with vacuolation, capillary proliferation, gliosis but relative neuronal preservation are hallmark findings [27]. In our patient, there was a clear pattern of astrogliosis, specifically involving the deeper layers of the cerebral cortex, which could be specific to this severe phenotype (as it transcribes in the animal model) or be a mere result of ATP depletion [27]. An interesting finding in our patient’s brain was the general reduction in the number of IBA1-positive microglia. In mitochondrial diseases, as well as in many other neuropathological conditions, microglia are often activated and part of a pathological response leading to neuronal death [28, 29]. In our patient, even in sites of pronounced astrogliosis indicating hypoxia and/or ATP-depletion, the lack of a microglial response was evident. To our knowledge, this has not been described previously in mitochondrial encephalopathies. Apart from being the brain’s scavengers, microglia are important in both pre- and postnatal brain development [29] by supporting neuronal survival, neurogenesis and oligodendrogenesis both in vitro [30, 31] and in vivo [32]. This has an impact on both plasticity and cognition [29], and we speculate that the severe neurological phenotype in our patient is, at least partially, due to the lack of microglia already *in utero*. Microglia are derived from a myeloid linage, present already in the yolk sac, from where they populate the brain rudiment in early embryogenesis [29, 33]. From thereon, they self-renew within the brain and thus are not replenished by circulating monocytes, unless there is an ongoing inflammatory condition. We further sought to investigate if other yolk sac-derived resident macrophages were affected [34] and found a clear reduction in KCs, the resident macrophages of the liver. These cells are important in many aspects of the liver function, including ischemia reperfusion injury and infectious disease [34]. However, our patient had no signs of severe liver disease until her death at 13 months of age. To our knowledge there are no descriptions in the literature of KC deficiency and its relation to disease. Why there is a specific lack of yolk sac-derived macrophages is unclear, however an *in situ* hybridization study showed that BCS1L is highly expressed in the yolk sac of mice [35] stressing its importance in early embryonic development.

Our patient showed severe hypotonia and a muscle biopsy revealed abnormal fibers, lipid inclusions, and aberrant mitochondria in electron microscopic analysis. These findings have not been seen in other BCS1L associated pathologies [4], and stress that novel mutations in mitochondrial genes can produce different phenotypes.

## Conclusions

In this report we present the genetic, biochemical and histological investigation of a patient with BCS1L deficiency and compare the histological findings to a mouse model. Apart from two previously not described mutations in the *BCS1L* gene (c.306A > T and c.399delA), and a thorough molecular biological and biochemical assessment to prove their pathogenicity, we also show novel histological findings, including aberrant muscle histology, a specific striped pattern of astrogliosis, and lack of microglia and KCs. This report points out the importance of an early thorough but focused genetic and biochemical investigation in order to diagnose these rare entities, requiring the combined effort of experts in several different fields.

## Additional files

Additional file 1: Figure S4. Transcript-specific primer design and verification of mis-splicing caused by c.306A > T. (A) Wild-type genomic sequence spanning *BCS1L* exons 3 and 4. The locations of the mutations identified in this study (c.306A > T in exon 3 and c.399delA in exon 4), the splice sites involved in exon 3 to 4 splicing, the predicted deletion caused by aberrant splicing at c.305, and the locations of the primers used in the mis-spliced allele-specific RT-PCR are shown. (B) Partial chromatogram from sequencing of the RT-PCR fragment amplified using primers specific for the predicted mis-spliced transcript caused by c.306A > T nucleotide change. This PCR product was amplified from the patient but not from the control fibroblast cDNA. (PDF 2123 kb)

Additional file 2: Figure S1. Muscle histology and electron microscopy. (A) NADH staining showing scattered fibers with enhanced reactivity. (B) Electron microscopy showing a fiber with increased amount of lipid droplets (L) and many mitochondria, some with structural abnormalities (arrow). Control muscle with normal mitochondria. Bars 2 μm. (PDF 4485 kb)

Additional file 3: Figure S2. Coronal section at the level of the left amygdala. The bulk of the white matter is reduced and shows discoloration in the temporal lobe. The corpus callosum is thin and there is moderate lateral and third ventricular dilation. Cortical laminar necrosis is seen in the cingulate gyrus, the superior frontal gyrus, the precentral gyrus, the inferior temporal gyrus and the lateral occipitotemporal gyrus (arrows). (PDF 5698 kb)

Additional file 4: Figure S3. BNGE with immunblotting. The samples were run on two gels in quadruplicate. (A). The gel was stained with commassie blue after blotting to PVDF membrane to show that the loading was similar; the first (lanes 2-5) and second (lanes 7-10) loading of the samples with the ladder (lanes 1 and 6) are shown. The molecular weights of the ladder markers are indicated. (B). For the upper blot the CORE1 and RISP antibodies were used, for the second blot the BCS1L antibody and the combination of CI NDUFVI (to detect the subunit assembled at the final stage), CIV Va, and CII 30kD were used, respectively. The first blot was stripped and thereafter the antibodies against CIV COX and CI NDUFA9 were probed (remnants of the CORE1 and RISP bands can be seen). Despite weaker bands in the patient (lanes 1 and 6) the decrease in BCS1L and RISP is recognizable. (PDF 2082 kb)

## Abbreviations

ATP: Adenosine triphosphate
BN PAGE: Blue Native PAGE
CI: Complex I
CII: Complex II
CIII: Complex III
CIV: Complex IV
CNVs: Copy number variations
COX: Cytochrome C oxidase
IVH: Intraventricular hemorrhage
KCs: Kupffer cells
OXPHOS: Oxidative phosphorylation
pre-CIII: Complex III pre-complex
qPCR: Quantitative polymerase chain reaction
RISP: Rieske iron-sulfur protein
