# Supplementary figures and images for: Respiratory chain complex III deficiency due to mutated BCS1L: a novel phenotype with encephalomyopathy, partially phenocopied in a Bcs1l mutant mouse model
Source: Orphanet J Rare Dis. 2017 Apr 20;12:73. doi: 10.1186/s13023-017-0624-2 (PMC5399415; doi:10.1186/s13023-017-0624-2)

A

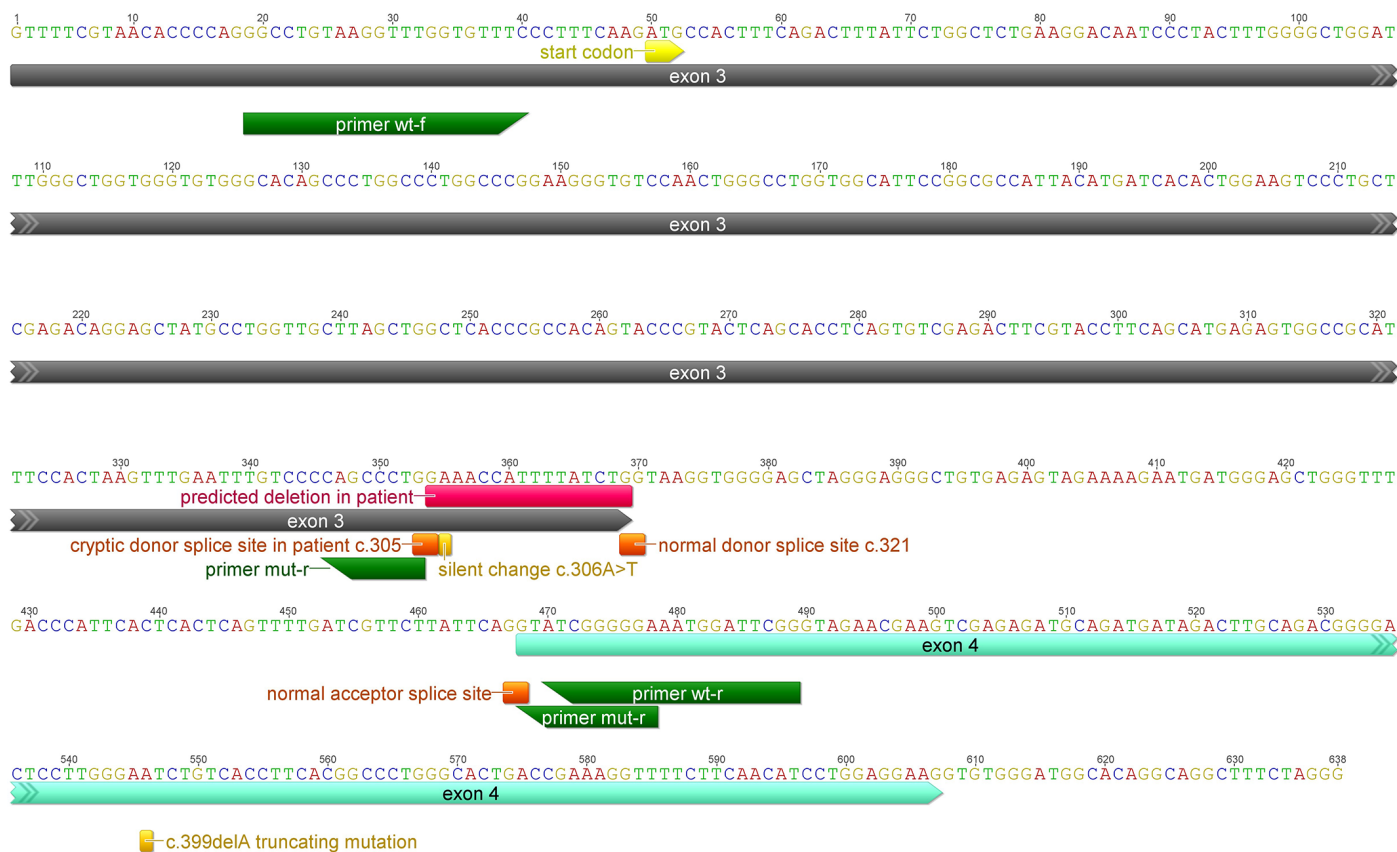

B

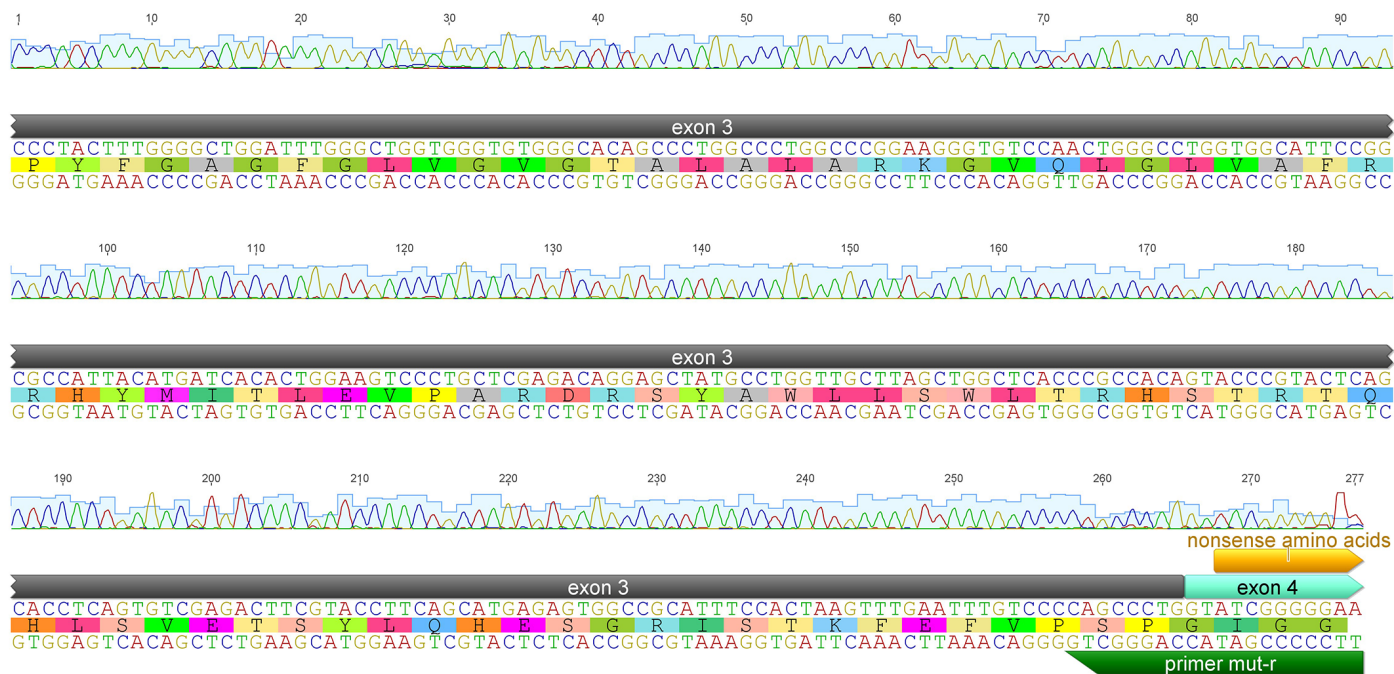

Supplement: Supplementary file 1 — Transcript-specific primer design and verification of mis-splicing caused by c.306A > T. (A) Wild-type genomic sequence spanning BCS1L exons 3 and 4. The locations of the mutations identified in this study (c.306A > T in exon 3 and c.399delA in exon 4), the splice sites involved in exon 3 to 4 splicing, the predicted deletion caused by aberrant splicing at c.305, and the locations of the primers used in the mis-spliced allele-specific RT-PCR are shown. (B) Partial chromatogram from sequencing of the RT-PCR fragment amplified using primers specific for the predicted mis-spliced transcript caused by c.306A > T nucleotide change. This PCR product was amplified from the patient but not from the control fibroblast cDNA. (PDF 2123 kb) [file 13023_2017_624_MOESM1_ESM.pdf]

A

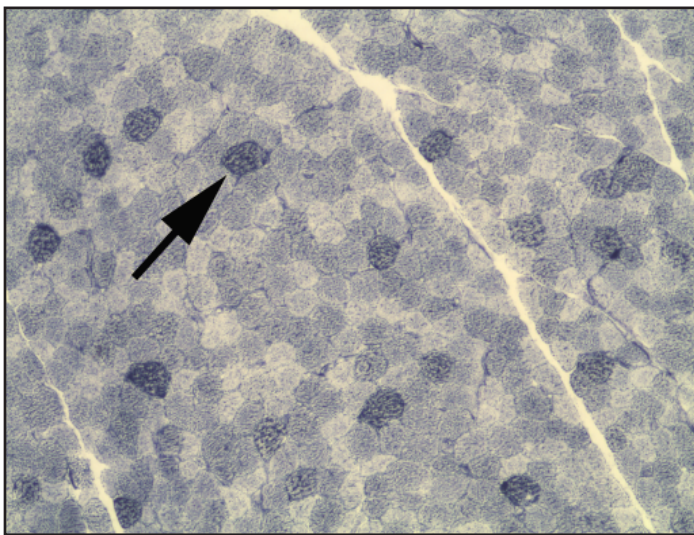

B

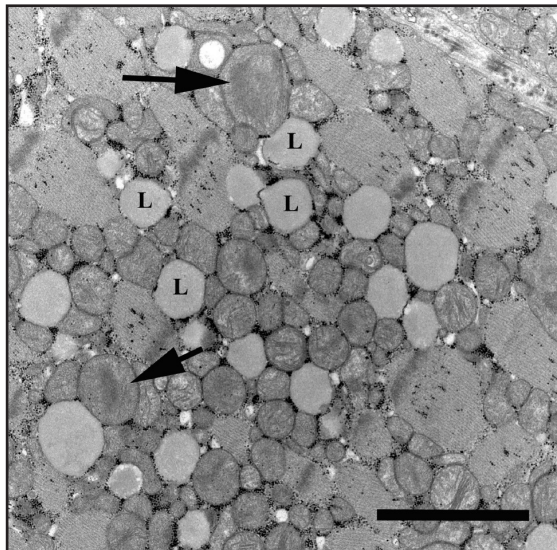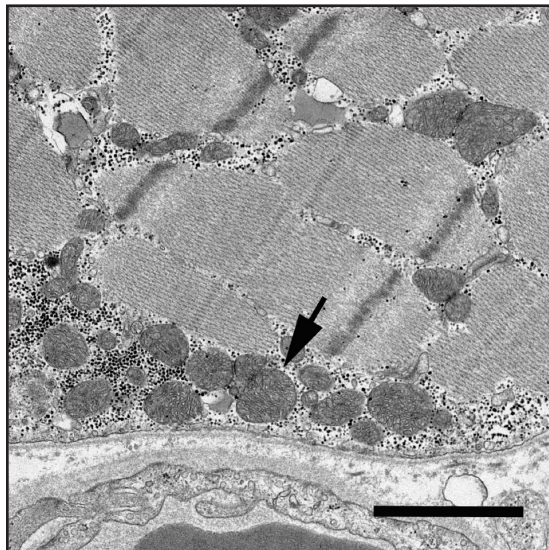

Supplement: Supplementary file 2 — Muscle histology and electron microscopy. (A) NADH staining showing scattered fibers with enhanced reactivity. (B) Electron microscopy showing a fiber with increased amount of lipid droplets (L) and many mitochondria, some with structural abnormalities (arrow). Control muscle with normal mitochondria. Bars 2 μm. (PDF 4485 kb) [file 13023_2017_624_MOESM2_ESM.pdf]

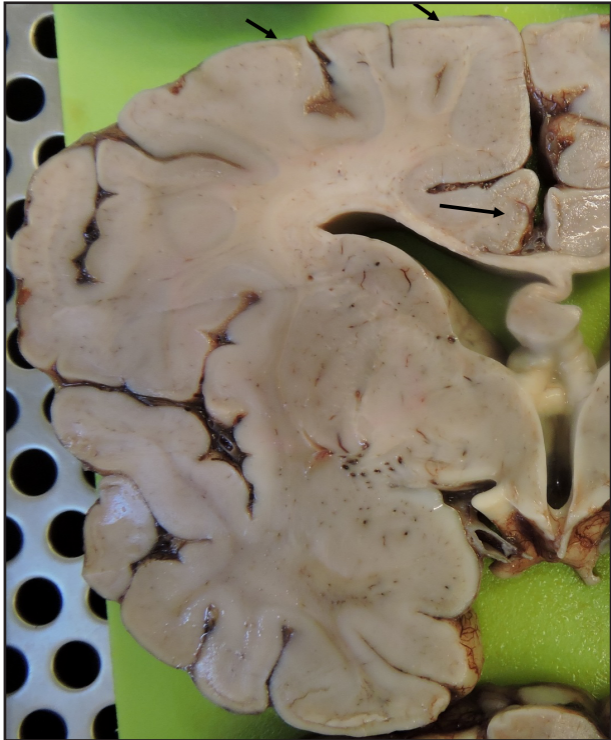

Supplement: Supplementary file 3 — Coronal section at the level of the left amygdala. The bulk of the white matter is reduced and shows discoloration in the temporal lobe. The corpus callosum is thin and there is moderate lateral and third ventricular dilation. Cortical laminar necrosis is seen in the cingulate gyrus, the superior frontal gyrus, the precentral gyrus, the inferior temporal gyrus and the lateral occipitotemporal gyrus (arrows). (PDF 5698 kb) [file 13023_2017_624_MOESM3_ESM.pdf]

# BNGE: Mitochondria

A

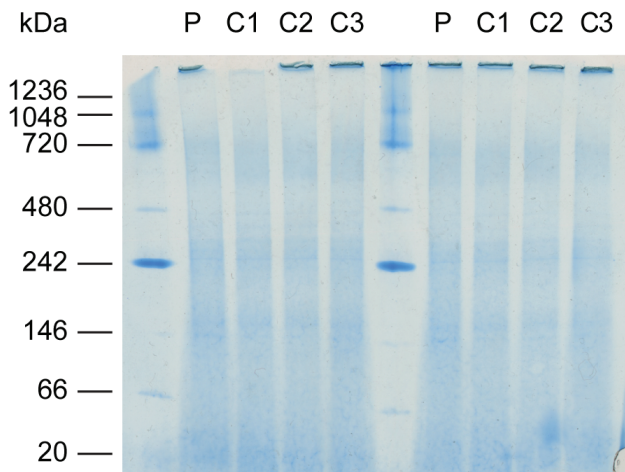

B

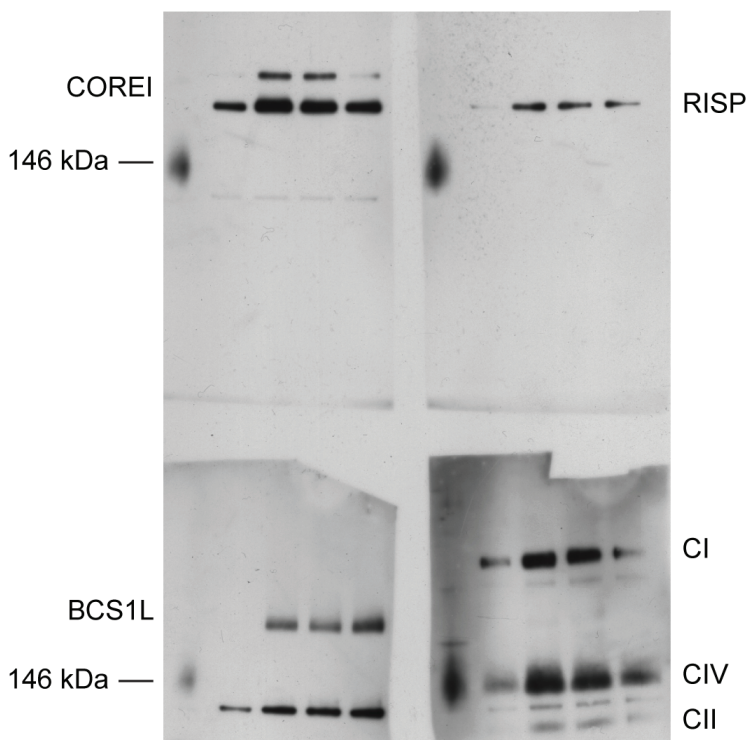

C

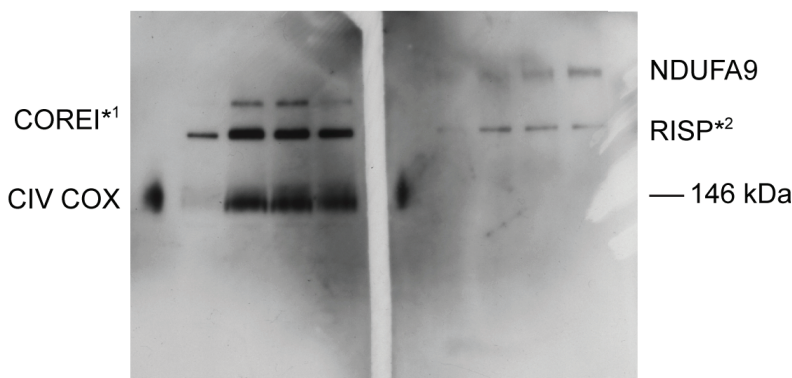

Supplement: Supplementary file 4 — BNGE with immunblotting. The samples were run on two gels in quadruplicate. (A). The gel was stained with commassie blue after blotting to PVDF membrane to show that the loading was similar; the first (lanes 2-5) and second (lanes 7-10) loading of the samples with the ladder (lanes 1 and 6) are shown. The molecular weights of the ladder markers are indicated. (B). For the upper blot the CORE1 and RISP antibodies were used, for the second blot the BCS1L antibody and the combination of CI NDUFVI (to detect the subunit assembled at the final stage), CIV Va, and CII 30kD were used, respectively. The first blot was stripped and thereafter the antibodies against CIV COX and CI NDUFA9 were probed (remnants of the CORE1 and RISP bands can be seen). Despite weaker bands in the patient (lanes 1 and 6) the decrease in BCS1L and RISP is recognizable. (PDF 2082 kb) [file 13023_2017_624_MOESM4_ESM.pdf]
